# Listening to the Silence: What does unrecorded information in the Electronic Health Record tell us? Findings from a Patient and Public Involvement Event

**DOI:** 10.23889/ijpds.v1i1.309

**Published:** 2017-04-19

**Authors:** Julie George, Serena Luchenski, Elizabeth Williamson, Amitava Banerjee, Peter E Saunders

**Affiliations:** Farr Institute of Health Informatics Research, UCL; London School of Hygiene and Tropical Medicine; PPI Forum, Farr Institute of Health Informatics Research, UCL

## Objectives

Handling missing information is an important methodological challenge in electronic health records research, with the reasons for missingness dictating how missing data should be handled. While there is extensive literature on methodological approaches to handling missing data, there is relatively little research with patients or clinicians investigating why information may be unrecorded in the medical records. A patient and public involvement event was held, embedded with a wider EHR event (UCL Festival of Digital Science - March 2016), to explore unrecorded information from a patient and researcher point of view with a view to developing further research in this area.

## Approach

Co-chaired by an academic and lay chair, members of the Farr Patient and Public Involvement Forum, academics, students and the general public attended workshop held during the Festival of Digital Health. A brief exercise was used to demonstrate the impact of missing data. Participants then worked in small facilitated groups to discuss situations where information about them (or people they knew) had not been recorded, the reasons why this information was not recorded and what the impact (or potential impact) was on the patient. A standardised data form was used by facilitators to record participants' conversations. The data forms were summarised into Excel and a brief thematic analysis was conducted. A written evaluation of the workshop was also completed by participants and included in the analysis.

## Results

The evaluation was overwhelming positive about embedding PPI workshop within an event with a wider remit, from both researchers and patient and public representatives. Preliminary findings on reasons for missingness included change of address or provider (both general practice and secondary care), individual clinician approaches to recording data, process of computerisation of medical notes, early stages of disease not reported to GP and unwillingness to disclose more sensitive information such as sexually transmitted disease. Discussion also included reasons for misrepresented or other errors with data. Further findings from the framework analysis will be presented.

## Conclusion

This exploratory workshop showed that patient and public involvement events can be embedded successfully within events aimed at professionals. Preliminary findings suggest that reasons for missingness often related to features of the provider or clinician as well as the patient.

